# Osteopontin promotes epithelial-mesenchymal transition of hepatocellular carcinoma through regulating vimentin

**DOI:** 10.18632/oncotarget.7016

**Published:** 2016-01-25

**Authors:** Qiongzhu Dong, Xuchao Zhu, Chun Dai, Xiaofei Zhang, Xiaomei Gao, Jinwang Wei, Yuanyuan Sheng, Yan Zheng, Jian Yu, Lu Xie, Yi Qin, Peng Qiao, Chuang Zhou, Xinxin Yu, Huliang Jia, Ning Ren, Haijun Zhou, Qinghai Ye, Lunxiu Qin

**Affiliations:** ^1^ Department of General Surgery, Huashan Hospital, Fudan University, Shanghai, China; ^2^ Liver Cancer Institute, Fudan University, Shanghai, China; ^3^ Institute of Biomedical Sciences, Fudan University, Shanghai, China; ^4^ Shanghai Center for Bioinformation Technology, Shanghai, China

**Keywords:** osteopontin, epithelial-mesenchymal transition, hepatocellular carcinoma, vimentin, stability

## Abstract

Our previous studies have found that osteopontin (OPN) is a promoter for hepatocellular carcinoma (HCC) progression. However, the molecular mechanism by which OPN enhances HCC metastasis remains elusive. Epithelial-mesenchymal transition (EMT) of cancer cells plays a pivotal role in promoting metastatic process. In this study, we demonstrated that OPN promotes HCC metastasis by inducing an EMT-like, more aggressive cellular phenotype *in vitro* and *in vivo*. Furthermore, OPN was identified to interact with vimentin by reciprocal OPN and vimentin immunoprecipitation as well as co-immunofluorescence examination. By using deletion mutants, we found that the residues between 246 and 406 in vimentin are required for binding to OPN. Importantly, OPN significantly increased vimentin stability through inhibition of its protein degradation. Knockdown of vimentin neutralized the EMT induced by OPN both *in vitro* and *in vivo*. Moreover, a significant correlation between OPN and vimentin levels was found in clinical HCC specimens and their combination had a worse prognosis with shorter overall survival (OS) and time to recurrence (TTR). In multivariate analysis, OPN and their combination were demonstrated to be independent prognostic indicators for OS and TTR of HCC patients. Collectively, this study indicates that OPN can induce EMT of HCC cells through increasing vimentin stability, which provides more in-depth understanding about the molecular mechanisms of OPN in promoting HCC metastasis and opens tantalizing therapeutic possibilities in HCC.

## INTRODUCTION

Hepatocellular carcinoma (HCC) is one of the most prevalent cancers and the third most frequent cause of cancer-related death worldwide [1]. Despite many progresses have been achieved in the clinical managements of HCC, its prognosis remains dismal [2]. The extremely poor prognosis of patients with HCC is largely due to the high rate of tumor recurrence or intrahepatic metastasis after surgical resection [3].

Metastasis, which consists of a series of discrete biological processes that move tumor cells from the primary neoplasm to underlying stroma, is responsible for as much as 90% of cancer-associated mortality [4]. Yet it remains the most poorly understood component of cancer pathogenesis. During the metastatic cascade, carcinoma cells often activate a key step known as epithelial-mesenchymal transition (EMT), a dynamic cellular process that facilitates tumor cells disseminate from the site of the primary tumor and establish secondary tumors in distant organs dissemination [5, 6]. EMT has been considered to play a prominent role in invasiveness and metastasis of various cancers including HCC [7]. Characteristic down-regulation of E-cadherin and increased expression of mesenchymal markers, such as N-cadherin, vimentin and fibronectin, are expected for cells undergoing EMT [8]. Snail and Twist, transcriptional repressors of E-cadherin as well as inducers of epithelial-mesenchymal transition [9], play pivotal roles in the development of tumor invasion and metastasis [10].

Osteopontin (OPN, also known as ETA1 and encoded by *SPP1*) plays a crucial role in tumor invasion and metastasis [11]. Our previous studies have shown that increased OPN levels are associated with poor prognosis of HCC [12, 13], and OPN is a promoter for HCC metastasis [14–16]. Thus, understanding how OPN are involved in maintaining aggressive HCC cell phenotypes may help identify novel potential targets for enhancing the efficacy of cancer therapeutics.

In this study, using protein interaction assays, and both *in vitro* and *in vivo* functional studies, we demonstrated that OPN induced EMT of HCC cells via increasing vimentin stability, which provides more in-depth understanding about the molecular mechanisms of OPN in promoting HCC metastasis and opens tantalizing therapeutic possibilities in HCC.

## RESULTS

### OPN could induce EMT of HCC cells

**Figure 1:**
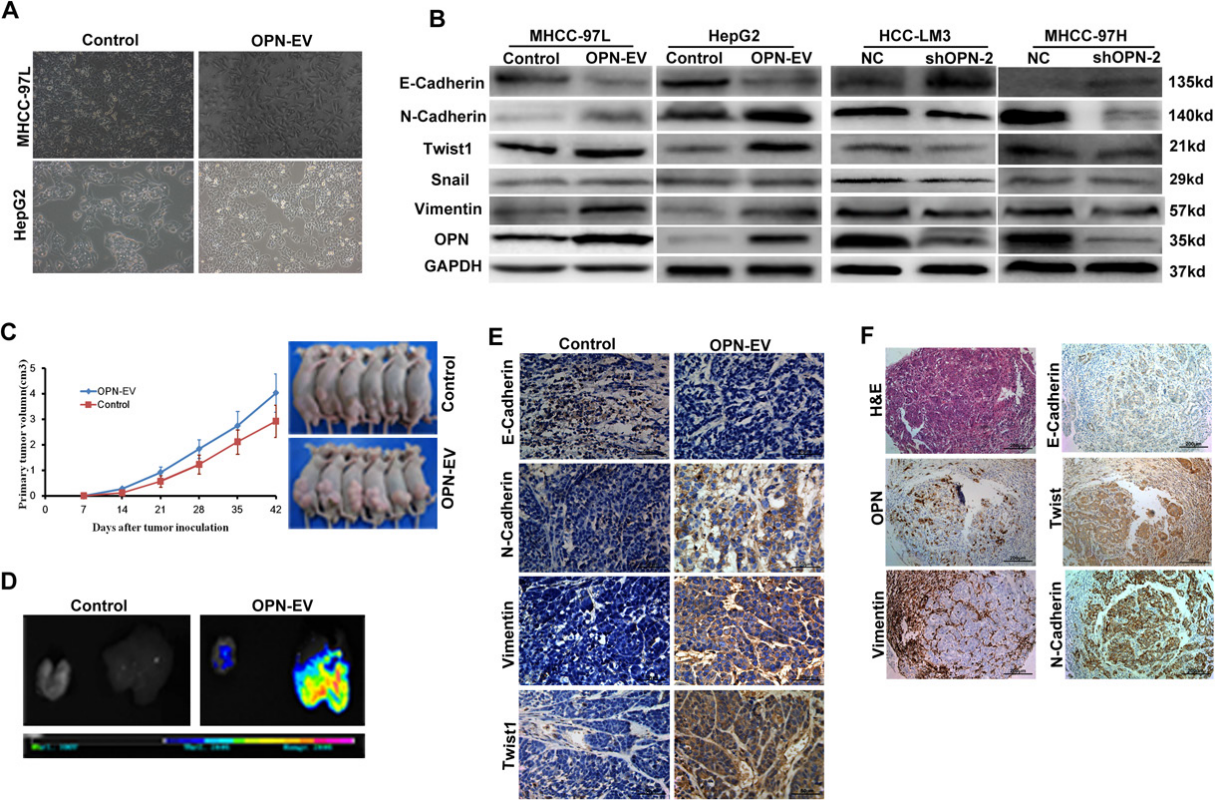
OPN promotes EMT in HCC cells (**A**) Representative pictures show the morphological change after overexpression of OPN in HCC cells. (**B**) Expression of EMT-related biomarkers were detected in MHCC-97L and HepG2 cells with up-regulation of OPN (left two panels) or HCC-LM3 and MHCC-97H cells with OPN knockdown (right two panels). (**C**) Both *in vivo* tumor growth rates (left panel) and tumor sizes (right panel) in mice models subcutaneously implanted with HepG2-OPN cells were much larger than that of those controls (*P* < 0.05). (**D**) The visual and fluorescent images demonstrated obviously stronger fluorescent signals in both liver and lung of nude mice models subcutaneously implanted with HepG2-OPN cells (right), while no obvious GFP signal was detected in liver or lungs of the controls (left). (**E**) Immunohistochemical staining for the expressions of EMT-related markers in subcutaneous xenografts tumor tissues from nude mice models of OPN-upregulated HepG2 cells and the controls (Magnification × 400. Bar = 50 μm). (**F**) Representative HCC cases in tissue slides (serial sections) were analyzed by H & E and immunohistochemical staining for OPN and EMT-related markers. (Magnification × 100. Bar = 200 μm).

In our previous studies, we found that OPN-specific antibody significantly inhibited lung metastasis of HCCLM3 cells [14] and blocking OPN expression suppressed growth and metastasis of HCC [15]. To further investigate the molecular mechanism by which OPN enhances HCC metastasis in this study, we stably overexpressed OPN in MHCC-97L and HepG2 cell lines, which are low metastatic [17] and have decreased levels of OPN [15, 18] (Supplementary Figure 1A). We found that up-regulation of OPN resulted in morphologic changes of HCC cells from the typical cobblestone-like appearance of epithelial cells to a spindle-like, fibroblastic morphology (Figure 1A). In consistent with the morphologic change, a decreased expression of epithelial marker E-cadherin concomitant with significant increases of mesenchymal markers including N-cadherin, vimentin, as well as the EMT major regulator Twist1 were found after OPN up-regulation (Figure 1B, left). In the other hand, knockdown of OPN in HCC-LM3 and MHCC-97H cell lines (Supplementary Figure 1B and 1C), which are high metastatic [17] and have increased levels of OPN [15, 18], induced an increase in E-cadherin level and significant decreases in the expression levels of N-cadherin, vimentin, and Twist1 (Figure 1B, right). But no significant alteration in Snail level was observed (Figure 1B).

In addition, up-regulation of OPN was demonstrated to significantly increase *in vitro* invasive (Supplementary Figure 2A), migrative abilities (Supplementary Figure 2B) and colony formation activity (Supplementary Figure 2C) of HCC cells, as assessed by the matrigel invasion chamber, wound healing assays and colony formation assays. To further test whether OPN overexpression induced EMT of HCC *in vivo*, we developed nude mice models with subcutaneous implantation of HCC cells. HCC cells with high-OPN level resulted in larger sizes of tumors and higher rates of metastasis when compared with the low-OPN HCC cells (Figure 1C and 1D). Of note, a significant correlation between OPN levels and EMT was found in the subcutaneous xenografts of nude mice models. Using immunohistochemistry assays, dramatic increases in N-cadherin, vimentin and Twist1 levels, and significantly decreased E-cadherin were demonstrated in subcutaneous xenografts of nude mice models bearing high-OPN HCC cells (Figure 1E). More importantly, HCC cells overexpressing OPN had a mesenchymal phenotype in human HCC tissues (Figure 1F). Taken together, these findings suggest that OPN overexpression promotes tumor progression by inducing EMT of HCC cells.

### Vimentin was identified to interact with OPN in HCC

**Figure 2:**
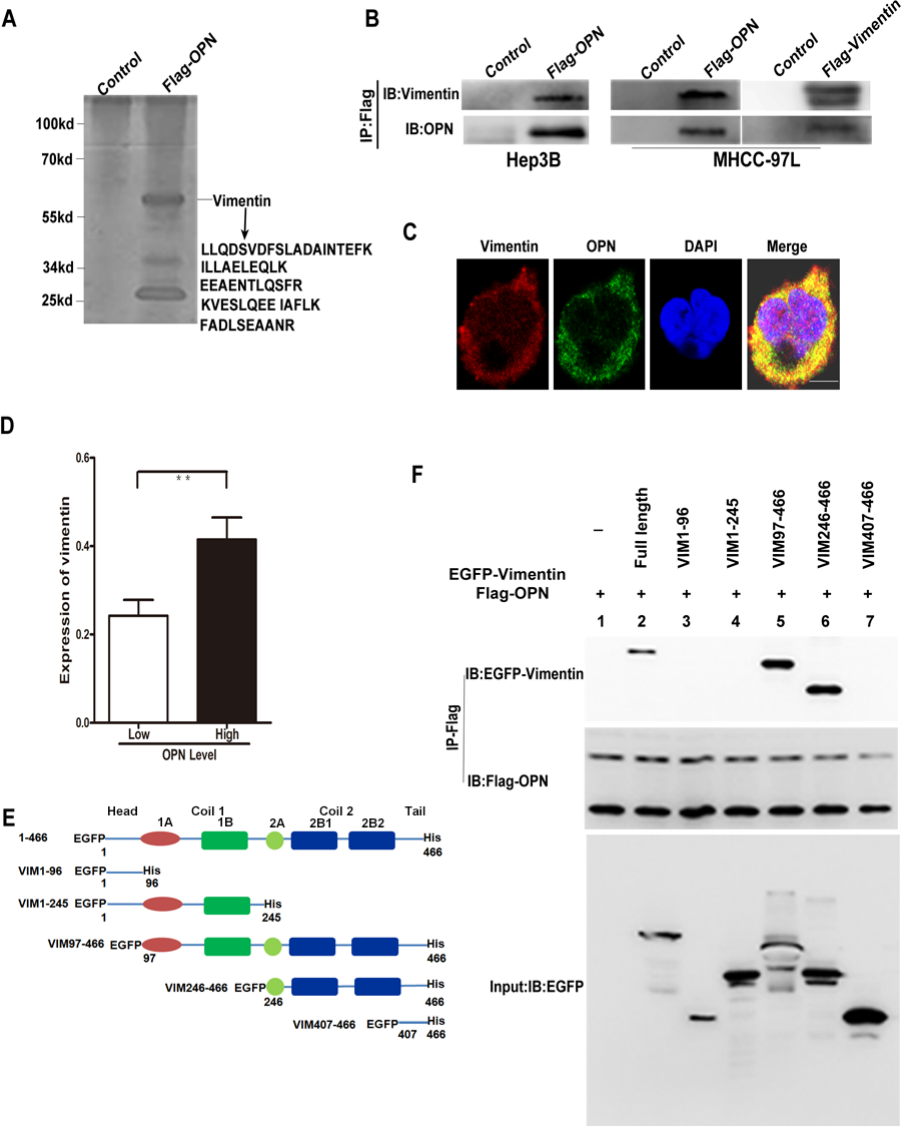
OPN binds to vimentin in HCC cells (**A**) Identification of OPN-associated factors using immunoprecipitation and mass spectrometry (IP/MS). Hep3B cells were transfected with Flag-OPN or empty vector. The purified OPN-associated proteins were detected by SDS-PAGE and Coomassie blue staining. Identified vimentin peptides are shown. (**B**) Co-IP assays showed OPN formed a complex with vimentin. HCC cells were transfected with Flag-OPN or Flag-vimentin and subjected to immunoprecipitation using anti-Flag mAb. HCC cells were transfected with empty vector used as control. Coimmunoprecipitated vimentin or OPN was detected using reciprocal antibodies respectively. (**C**) Colocalization of OPN (green) and vimentin (red) in MHCC-97L cells by immunofluorescence. Nuclei were stained with DAPI. (Bar = 20 μm). (**D**) OPN and vimentin expression are positively correlated in HCC tissues (**P < 0.01). (**E**) Diagrammatic representation of vimentin and its deletion mutants. (**F**) Co-IP assays were performed from cell lysates transfected with full length vimentin or its deletion mutants. Immunoprecipitation was carried out with anti-Flag (against OPN). Immunoblot analysis was performed with the indicated antibodies.

To determine how OPN to induce EMT, a combination of co-IP and MS was used to identify the interactome of OPN in Hep3B-OPN and Hep3B control cells. Flag-OPN produced in Hep3B cells was immunoprecipitated by anti-Flag mAb and coprecipitated proteins were visualized by *Coomassie blue staining* after electrophoresis and identified by Mass spectrometry (MS) (Figure 2A). One candidate OPN-interacting protein in this search was vimentin, of which five peptides, LLQDSVDFSLADAINTEFK, ILLAELEQLK, EEAENTLQSFR, KVESLQEE IAFLK and FADLSE AANR, were identified (Supplementary Figure 3). Vimentin, a mesenchymal-related protein, functionally contributes to EMT [19]. To confirm the interaction between OPN and vimentin, immunoprecipitation (IP) using anti-OPN antibodies revealed the presence of vimentin by immunoblotting in Hep3B-OPN cells (Figure 2B, left). Similarly, reciprocal co-IP experiments also showed that OPN was co-precipitated with vimemtin in MHCC-97L cells expressing high levels of OPN or vimentin (Figure 2B, right).

Moreover, confocal microscopy demonstrated that OPN and vimentin were co-localized in the cytoplasm of four HCC cell lines (Figure 2C, Supplementary Figure 4). We next determined the expression of OPN and vimentin protein in 374 HCC tissues and analyzed the relationship of both molecules by immunohistochemistry and tissue microarrays. Obviously, vimentin levels were closely correlated to OPN expression levels in HCC tissues (*P* < 0.001) (Figure 2D).

### Mapping the binding domains of vimentin to OPN

Next, to gain more insights on the OPN-vimentin interaction, the regions of vimentin responsible for their binding were mapped. A schematic representation of the full-length human vimentin (residues 1-466) and of the five deletion mutants were shown in Figure 2E. Vimentin deletion mutant VIM1-96 contains only head domain, VIM1-245 contains head domain and alpha-helices 1A-B, VIM97-466 mutant lacks the head domain, VIM407-466 contains the tail domain [20] (Figure 2E). To identify the region of vimentin that binds to OPN, we transfected a series of constructs encoding EGFP tagged vimentin deletion mutants together with OPN-Flag construct into MHCC97L cells. The cell lysates were subjected to IP using anti-Flag beads. The beads were then precipitated, with half being subjected to immunoblot analysis with anti-EGFP and anti-Flag. As expected, the endogenous full-length vimentin was binded OPN within HCC cell (Figure 2F). Among all five vimentin fragments, only the VIM97-466 and VIM 246-466 fragments were capable of binding to OPN. But deletion mutant of VIM 407-466 could not bind to OPN (Figure 2F). Collectively, the results indicate that vimentin binds to OPN at the region between residues 246 and 406. Furthermore, *in vitro* binding assay experiment showed that OPN bound only vimentin deletion mutants containing the central rod domain (VIM97-466 and VIM 246-466; Supplementary Figure 5). Deletion of the rod domain was sufficient to abolish binding of vimentin to OPN (Supplementary Figure 5). These results suggest that the central rod domain from alpha-helices 2A-B of vimentin is required for its binding to OPN.

### OPN increased the stability of vimentin

**Figure 3:**
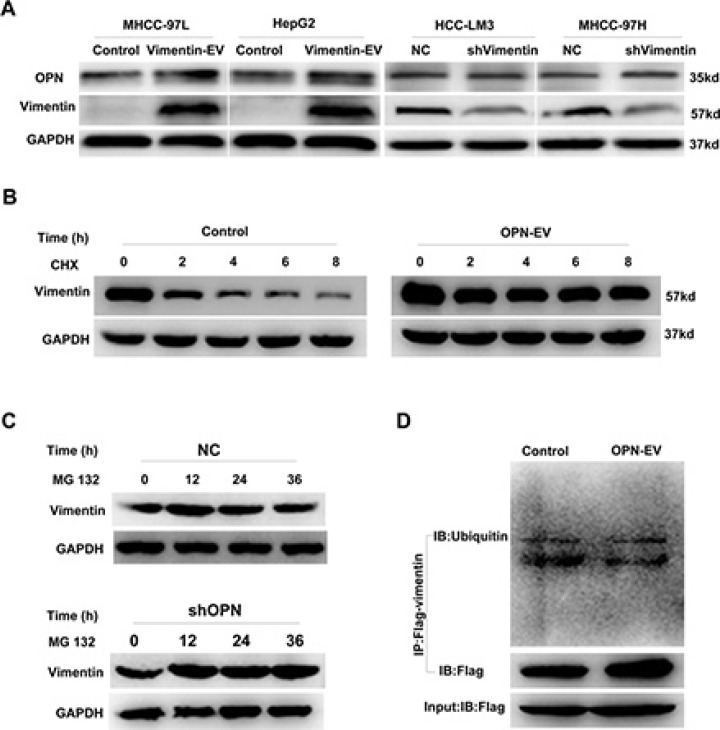
OPN increased the stability of vimentin (**A**) Overexpression (left two panels) or knockdown (right two panels) of vimentin in HCC cells showed little influence on OPN expression. (**B**) MHCC-97L cells with stable overexpression of OPN were treated CHX (20 μg/mL) for indicated times. Endogenous vimentin protein levels were determined. MHCC-97L cells were stably transfected with empty vector used as control. (**C**) MHCC-97H cells transfected with shOPN or scrambled shRNA were treated MG-132 (40 μM) for indicated times. Endogenous vimentin protein levels were determined. (**D**) Overexpression of OPN attenuates vimentin ubiquitin. Vector control and OPN stably overexpressing MHCC-97H cells were transfected with FLAG-tagged vimentin plasmids for 24 h and treated with 40 μM MG132 for 36 hours. Cells were then harvested and lysed immunoprecipitation using anti-Flag beads.

In the aforementioned cells, up- or down-regulation of OPN resulted in a corresponding increase or decrease of vimentin protein level (Figure 1B). However, up- or down-regulation of vimentin had no significant effect on OPN expression (Figure 3A), respectively. Thus, we hypothesized that the interaction between OPN and vimentin affects the steady-state level of vimentin. We next sought to determine whether the half-life of vimentin differed in the presence of OPN. To test this, we treated HCC cells with a protein synthesis inhibitor, cycloheximide (CHX), at different time points and measured the half-life of endogenous vimentin. As shown in Supplementary Figure 6 and Figure 3B, vimentin had a short half-life of < 4 hours (h), whereas up-regulation of OPN dramatically decreased the rate of vimentin degradation, resulting in a half-life of more than 8 h. In the cells which OPN expression was inhibited by shOPN, vimentin degradation was further increased at the time points (Supplementary Figure 7). Therefore, OPN-dependent stabilization is attributed to elongate the half-life of vimentin, leading to an increase in the amount of vimentin.

In the presence of the proteasomal inhibitor MG132, the reduced endogenous vimentin protein caused by shOPN was restored to the level comparable to that of the scrambled shRNA (Figure 3C). These findings suggest that the degradation of vimentin involves the typical proteasome-mediated pathway. Since ubiquitination is a potential mechanism underlying vimentin proteasome degradation, our attempts to show vimentin ubiquitination used reciprocal IP with anti-vimentin in HCC cells expressing high levels of OPN or empty control and then subjected to Ubiqitin IB. Upon OPN upregulation, the level of ubiquitination of vimentin was downregulated (Figure 3D). These data indicate that OPN may play an important role in suppression of the vimentin protein degradation.

### Vimentin is essential for OPN to induce EMT in HCC

**Figure 4:**
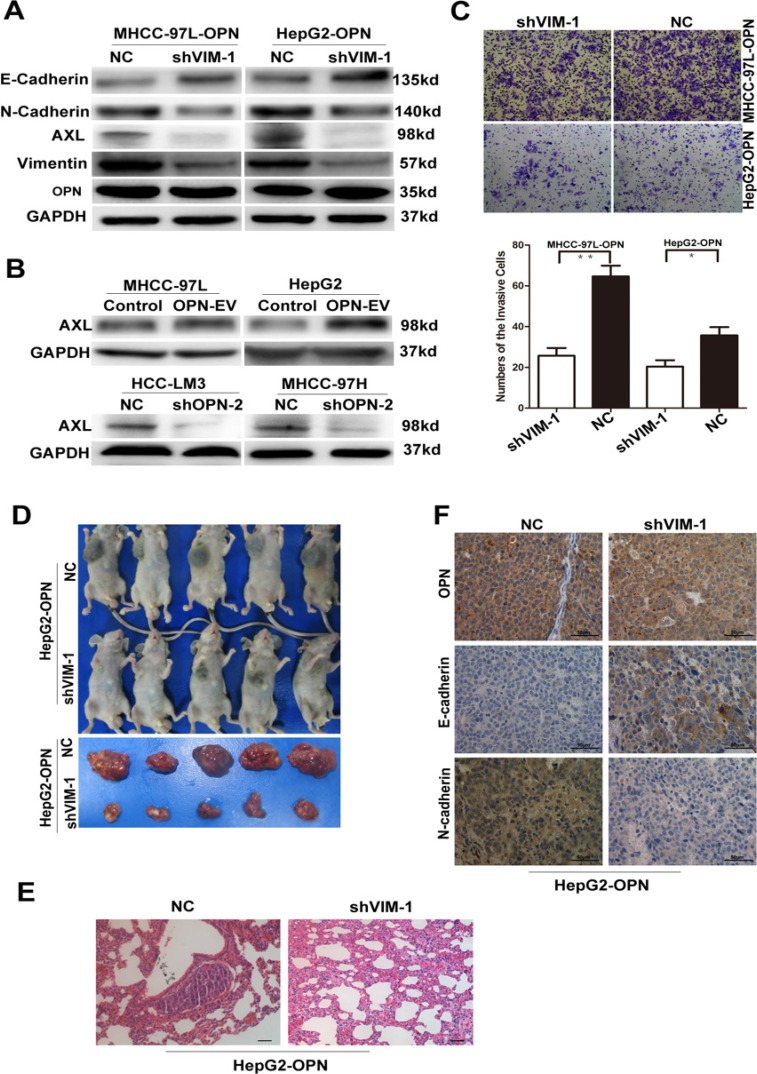
Vimentin is essential for OPN-induced EMT in HCC (**A**) EMT-related markers were detected in MHCC-97L-OPN and HepG2-OPN cells transfected with shVimentin or scrambled shRNA. (**B**) Expression of Axl were determined in MHCC-97L and HepG2 cells with up-regulation of OPN (upper) or HCC-LM3 and MHCC-97H cells with OPN knockdown (lower). (**C**) The *in vitro* invaded cell numbers were analyzed in MHCC-97L and HepG2 cells with stable overexpression of OPN transfected with shVimentin or scrambled shRNA. *P < 0.05, **P < 0.01. (**D**) The tumor volumes derived from HepG2-OPN cell lines stably transfected with shVimentin or scrambled shRNA was measured *in vivo* for 6 weeks. (**E**) Serial sections from mouse lungs showed the metastatic ability of HepG2-OPN cell lines stably expressing different levels of vimentin (Bar = 50 μm). (**F**) Immunohistochemical staining for the expressions of EMT-related markers in subcutaneous xenografts tumor tissues from nude mice models. (Magnification × 400. Bar = 50 μm).

Vimentin is a hallmark of primary tumor progression to a metastatic phenotype, which may be involved in the modulation of EMT [19]. To identify whether vimentin is involved in OPN-induced enhancing EMT, we assessed EMT markers in OPN-upregulation HCC cells transfected with shVimentin or scrambled shRNA (Supplementary Figure 8). The reduced level of vimentin led to the reversal of the EMT phenotype in OPN-upregulation HCC cells, with the increased of E-cadherin and decreased N-cadherin levels (Figure 4A).

Moreover, we examined the expression of Axl, a key determinant of cell migration and EMT promotion increased by vimentin [21]. Interestingly, up- or down-regulation of OPN also induced increased or decreased Axl expression levels in parallel with corresponding vimentin levels in HCC cells (Figure 4B). However, Axl was significantly down-regulated in OPN-upregulation HCC cells when vimentin expression was suppressed by RNA interference (Figure 4A).

Because cells that undergo EMT often gain migratory and invasive capabilities, we tested the invasion ability of these cells. We further found that the synergistic effect of OPN on HCC cell invasion was antagonized by silencing of vimentin (Figure 4C).

To further determine whether vimentin is involved in OPN-induced enhancing EMT *in vivo*, HepG2-OPN cells stably transfected with shVimentin or scrambled shRNA were injected subcutaneously into nude mice. We found that HepG2-OPN cells transfected with shVimentin displayed smaller tumors when compared with HepG2-OPN cells transfected with scrambled shRNA (Figure 4D). The incidence of pulmonary metastasis was 60% (3/5) in the group of HepG2-OPN cells transfected with scrambled shRNA, but no any lung metastasis was found in the group of HepG2-OPN cells transfected with shVimentin (Figure 4E).

In addition, we investigate the expression of EMT markers in subcutaneous models. Staining intensities for N-cadherin were greatly decreased in HCC tissues from subcutaneous implantation models of HepG2-OPN cells stably transfected with shVimentin compared with controls, accompanied by increased E-cadherin expression (Figure 4F). These data provide further support that OPN increases metastasis through activation of vimentin-induced EMT response in HCC Cells.

### Combination of OPN and vimentin exhibits improved prognostic accuracy for HCC

To evaluate the possible roles of vimentin in HCC, we further investigated the expression levels of vimentin in human HCC tissues and various HCC cell lines. Immunohistochemical analyses demonstrated markedly increased vimentin levels in tumor tissues compared to their paired noncancerous liver tissues (Supplementary Figure 9A). Moreover, vimentin expression in high-metastatic HCC cell lines was much higher than that of the low-metastatic HCC cell lines both at the protein and mRNA levels (Supplementary Figure 9B).

**Figure 5:**
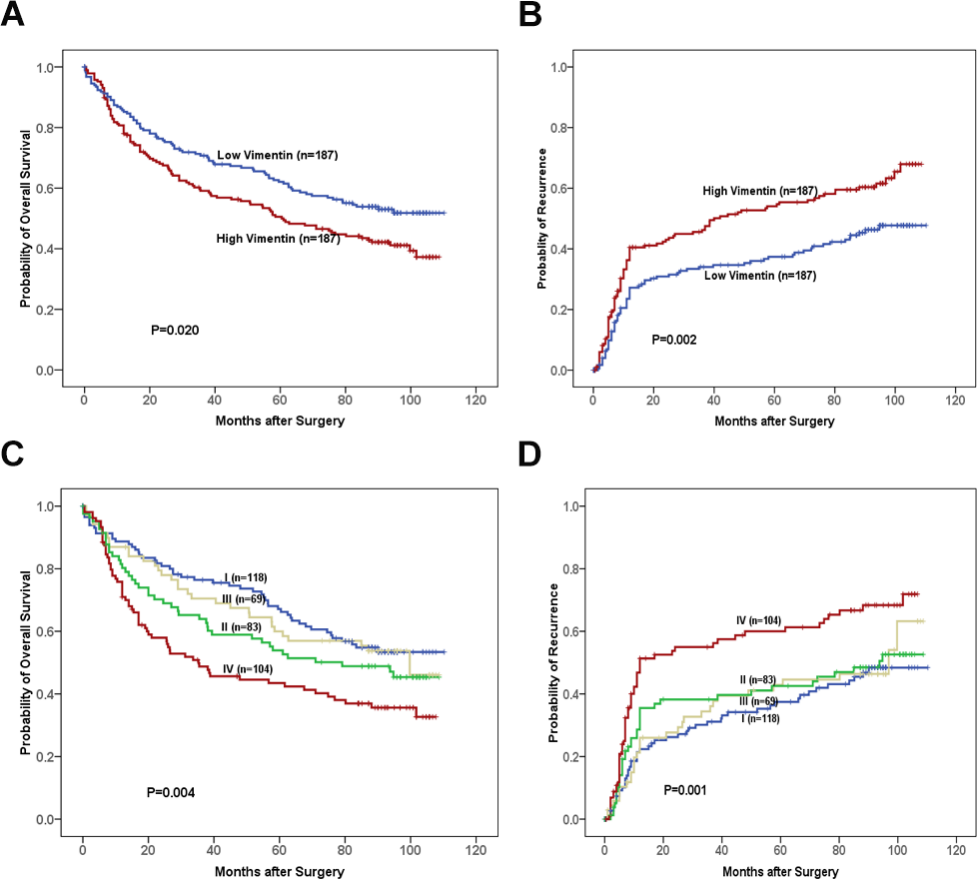
The prognostic significances of vimentin and OPN for HCC patients assessed by Kaplan-Meier analyses A, B. The associations of vimentin levels in HCC tissues with overall survival (OS) rates (**A**) and probabilities of tumor recurrence (**B**) of patients after HCC resection. C, D. The differences in OS rates (**C**) and probabilities of tumor recurrence (**D**) among the four different subgroups based on the combinations of OPN and vimentin, i.e., I, low-OPN/low-vimentin; II, high-OPN/low-vimentin; III, low-OPN/high-vimentin; and IV, high-OPN/high-vimentin. For each cohort, different subgroups were plotted according to the cut-off values of OPN and vimentin level which were defined as the median of the cohort. The patients in subgroup I had the longest OS and the lowest possibility of tumor recurrence.

The clinical significances of vimentin and OPN for HCC were further investigated using tissue microarrays containing HCC tissues from 374 patients and immunohistochemistry staining. The results revealed that higher protein levels of vimentin were significantly associated with ALT (*P* = 0.041) and tumor differentiation (*P* < 0.001) (Table 1). The overall survival (OS) rates of HCC patients with high vimentin were obviously lower than that of those low vimentin patients (*P* = 0.020, Figure 5A). In addition, the tumor recurrence rates of high-vimentin group were significantly higher than that of those with low vimentin (*P* = 0.002, Figure 5B).

**Table 1:** Association of OPN and vimentin expression levels with clinicopathologic characteristics of HCC patients

Variables	OPN			Vimentin		
	Low expression (*n* = 187)	High expression (*n* = 187)	*P* Value	Low expression (*n* = 187)	High expression (*n* = 187)	*P* Value
Gender						
Female	31	37	0.503	32	36	0.688
Male	156	150		155	151	
Age (years)						
≤ 50	96	91	0.679	94	93	1.000
> 50	91	96		93	94	
HBsAg						
No	19	7	**0.024**	16	10	0.309
Yes	168	180		171	177	
ALT						
≤ 75 U/L	170	165	0.499	174	161	**0.041**
> 75 U/L	17	22		13	26	
Liver cirrhosis						
No	20	22	0.870	21	21	1.000
Yes	167	165		166	166	
AFP (ng/ml)						
≤ 20	73	65	0.453	78	60	0.068
> 20	114	122		109	127	
Tumor size						
≤ 5 cm	142	152	0.256	143	151	0.377
> 5 cm	45	35		44	36	
Tumor number						
Single	176	180	0.470	177	179	0.810
Multiple	11	7		10	8	
Tumor capsule						
Complete	102	99	0.836	110	91	0.062
none	85	88		77	96	
Vascular invasion						
No	140	120	**0.033**	129	131	0.911
Yes	47	67		58	56	
Tumor differentiation						
I∼II	157	123	**< 0.001**	156	124	**< 0.001**
III∼IV	30	64		31	63	
BCLC stage						
0 and A	45	52	0.479	46	51	0.637
B and C	142	135		141	136	

Note: HBsAg, hepatitis B surface antigen; ALT, alanine amino transferase; AFP, alpha-fetoprotein; BCLC stage, Barcelona Clinic Liver Cancer stage.

A “low” versus “high” OPN and vimentin expression was defined according to the cut-off values of OPN and vimentin level which were defined as the median of the cohort.

All analysis was conducted using the Spearman rank and Fisher's exact tests.

In consistent with our previous study in HCC tissue [18, 22], we found that OPN level in tumor tissues was significantly associated with HBsAg (*P* = 0.024), vascular invasion (*P* = 0.033), and tumor differentiation (*P* < 0.001) (Table 1), as well as prognosis of HCC patients (Supplementary Figure 10A and 10B). In addition, the expression levels of OPN were significantly up-regulated in HCC tissues compared with their adjacent non-tumor liver tissues (*P* < 0.001, Supplementary Figure 11A). Moreover, the expression levels of OPN were significantly higher in metastatic HCC tissues (with intrahepatic spreading, or tumor invasion in blood vessel or bile duct) than the non-metastatic HCC tissues (*P* < 0.05) (Supplementary Figure 11B). Similarly, the levels of OPN were much obviously increased in the recurrent group (patients had intrahepatic tumor recurrence or extrahepatic metastasis during follow-up after operation) in comparison to the non-recurrent group (*P* < 0.05) (Supplementary Figure 11C). Tumor thrombi in portal vein (PVTT) from HCC notably deteriorates hepatic function and serves as a poor prognostic factor associated with frequent recurrence and intrahepatic metastasis (17). To define the specific changes associated with metastatic progression, we analyzed an additional 10 pairs of HCC samples with quantitative real-time polymerase chain reaction (qRT-PCR) analysis, and found that OPN levels were low in nontumorous tissue, relatively higher in primary HCCs by 4.5-fold and further increased in PVTT by 11.7-fold (Supplementary Figure 11D).

To further evaluate the prognostic values of the combination of OPN and vimentin, we divided patients into four groups based on OPN and vimentin expression levels. HCC patients with high OPN and high vimentin had the poorest prognosis with the lowest OS and highest probability of tumor recurrence. In contrast, those with low OPN and low vimentin had the best OS, lowest time to recurrence (TTR), and best prognosis (*P* = 0.004, *P* = 0.001; respectively) (Figure 5C and 5D). The combination of OPN and vimentin levels was an independent prognostic indicator for both OS (*P* = 0.029) and TTR (*P* = 0.007), which had a better prognostic value than OPN or vimentin alone (Table 2).

**Table 2:** Univariate and multivariate analyses of factors associated with OS and TTR of HCC (*n* = 374)

Variable	OS			TTR		
	HR	95% CI	*P* value	HR	95% CI	*P* value
**Univariate analysis**						
OPN (High vs. Low)	1.54	1.16–2.06	0.003	1.51	1.12–2.03	0.007
Vimentin (High vs. Low)	1.40	1.05–1.87	0.021	1.58	1.18–2.13	0.002
Sex (female vs. male)	1.04	0.72–1.50	0.848	1.08	0.73–1.59	0.710
Age, years (> 50 vs. ≤ 50)	1.19	0.90–1.59	0.226	1.09	0.81–1.46	0.563
ALT (> 75 vs. ≤ 75 U/L)	1.20	0.77–1.85	0.418	1.00	0.62–1.61	0.993
AFP, ng/mL (> 20 vs. ≤ 20)	1.60	1.18–2.18	0.003	1.41	1.03–1.91	0.030
Liver cirrhosis (yes vs. no)	1.33	0.81–2.19	0.263	1.48	0.88–2.47	0.138
HBsAg (positive vs. negative)	1.61	0.85–3.05	0.142	3.42	1.41–8.32	0.007
Tumor size, cm (> 5 vs. ≤ 5)	1.63	1.18–2.26	0.003	1.49	1.06–2.11	0.023
Tumor number (multiple vs. single)	1.98	1.13–3.49	0.017	1.26	0.64–2.47	0.500
Tumor capsule (none vs. complete)	1.41	1.06–1.88	0.017	1.32	0.99–1.77	0.062
Vascular invasion (yes vs. no)	1.99	1.49–2.67	< 0.001	1.65	1.21–2.26	0.002
BCLC stage (B and C vs. 0 and A)	1.81	1.27–2.58	0.001	1.47	1.04–2.07	0.029
Tumor differentiation (III∼IV vs. I∼II)	1.54	1.12–2.10	0.007	1.42	1.02–1.97	0.038
Combination of OPN and vimentin			0.005			0.002
II vs. I	1.30	0.87–1.95	0.182	1.15	0.75–1.77	0.514
III vs. I	1.08	0.69–1.68	0.572	1.06	0.68–1.67	0.789
IV vs. I	1.86	1.29–2.68	0.001	1.94	1.33–2.83	0.001
**Multivariate analysis^a^**						
OPN (High vs. Low)	1.42	1.05–1.93	0.023	1.39	1.02–1.90	0.037
Vimentin (High vs. Low)	1.29	0.95–1.73	0.101	1.51	1.11–2.05	0.010
AFP, ng/mL (> 20 vs. ≤ 20)	1.39	1.01–1.92	0.042	1.22	0.89–1.69	0.216
Tumor size, cm (> 5 vs. ≤ 5)	1.43	1.01–2.03	0.042	1.37	0.95–1.99	0.093
Vascular invasion (yes vs. no)	1.52	1.09–2.11	0.013	1.39	0.98–1.98	0.067
BCLC stage (B and C vs. 0 and A)	1.41	0.94–2.12	0.097	1.24	0.84–1.84	0.282
Tumor differentiation (III∼IV vs. I∼II)	1.15	0.82–1.60	0.422	1.05	0.74–1.50	0.781
**Multivariate analysis^b^**						
AFP, ng/mL (> 20 vs. ≤ 20)	1.40	1.02–1.93	0.036	1.25	0.91–1.72	0.177
Tumor size, cm (> 5 vs. ≤ 5)	1.42	1.00–2.01	0.049	1.34	0.93–1.95	0.121
Vascular invasion (yes vs. no)	1.51	1.09–2.11	0.014	1.39	0.97–1.97	0.070
BCLC stage (B and C vs. 0 and A)	1.41	0.94–2.12	0.101	1.23	0.83–1.82	0.313
Tumor differentiation (III∼IV vs. I∼II)	1.14	0.82–1.60	0.435	1.05	0.74–1.50	0.794
Combination of OPN and vimentin			0.029			0.007
II vs. I	1.36	0.90–2.05	0.148	1.18	0.77–1.82	0.448
III vs. I	1.15	0.73–1.80	0.545	1.11	0.70–1.76	0.655
IV vs. I	1.78	1.20–2.63	0.004	1.91	1.28–2.85	0.002

Univariate analysis, Cox proportional hazards regression; Multivariate analysis, Cox proportional hazards regression; Variables were adopted in multivariate analysis for their prognostic significance by univariate analysis.

A “low” versus “high” OPN and vimentin expression was defined according to the cut-off values of OPN and vimentin level which were defined as the median of the cohort.

Abbreviations: CI, confidence interval; HR, hazard ratio; OS, overall survival; TTR, time to recurrence.

I, Low-OPN and Low-vimentin; II, high-OPN and low-vimentin; III, low-OPN and high-vimentin; IV, High-OPN and High-vimentin.

a Multivariate analysis of OPN, vimentin, AFP, Tumor size, Vascular invasion, BCLC stage and Tumor differentiation.

b Multivariate analysis of Combination OPN and vimentin, AFP, Tumor size, Vascular invasion, BCLC stage and Tumor differentiation.

## DISCUSSION

Great efforts have been made to elucidate the molecular mechanism underlying tumorigenicity, invasion and metastasis of cancer in order to develop novel treatments and a possible cure in the past several decades.

Many previous works from our group and others have suggested that OPN plays important roles in tumor development, invasion and metastasis of HCC [12–14, 16, 23]. Blocking OPN expression was able to suppress growth and metastasis of HCC [15, 24]. But the mechanisms underlying the effects of OPN in promoting metastasis are not clearly understood yet. Our current study reinforces the notion that OPN is an important promoter for HCC metastasis, and more important, we found OPN is a pivotal modulator of the molecular and functional characteristics of EMT through regulating vimentin.

Metastases represent the end results of a multistep cell-biological process. The EMT appears to be important for cancer cells to acquire the capability of migration and invasion [7]. In this study, we found that OPN overexpression was significantly correlated with up-regulation of the mesenchymal markers and E-cadherin down-regulation. Using a mouse model, we also present *in vivo* evidences that OPN strongly increases the metastatic potential of HCC cells along with changes in their expression pattern of EMT markers. These results were further confirmed by immunohistochemical staining of clinical HCC specimens. Interestingly, recent studies showed that OPN was found to activate EMT-associated growth of HCC in a mouse xenograft model [25] and lead to EMT of hepatocytes in HCV-associated HCC [26]. These are in line with our data, which strongly suggest that OPN promotes HCC metastasis via, at least in part, inducing EMT of HCC cells.

A great number of factors and signals have been described to contribute to EMTs of cancer cells [27, 28]. By MS with subsequent co-IP and immunofluorescent staining, we for the first time demonstrated that OPN interacted with vimentin. Mapping of vimentin OPN-binding region showed the central rod domain from alpha-helices 2A-B of vimentin is required for its binding to OPN. Furthermore, this close correlation was confirmed in tumor tissues from a large cohort of HCC patients, and the combination of OPN and vimentin had a better prognostic performance than OPN or vimentin alone. These indicate that the concerted activities of OPN and vimentin detected in our experiments are recapitulated in clinical patients with HCC.

Proteolysis represents an important mechanism that controls protein concentration and function. It has been reported that OPN associated with protein degradation in murine mammary epithelial tumor cells [29] and human brain [30]. Our study reveals that OPN could increase the stability of vimentin through elongating its half-life. Although our present results demonstrated that OPN targets vimentin for degradation via the the ubiquitin-proteasome pathway, it should also be noted that there was no evidence for the typical ladder for vimentin ubiquitination–which are consistent with the recent report [31]. Interestingly, a recent study showed that an RNA aptamer (APT) which binds secreted OPN in the extracellular space significantly decreased vimentin expression [25] and knockdown of OPN resulted in decreased vimentin expression in in glioma cells [32]. The consistency between OPN and vimemtin suggests a possible common strategy that OPN may increase the expression of vimentin.

Vimentin, a major constituent of the intermediate filament (IF) family of proteins, is a marker of mesenchymal cells that is not expressed in epithelial cells [33]. Recently vimentin is considered as one of the EMT markers, and as a prerequisite for EMT induction [19]. It has been found to correlate with metastasis of various cancers including HCC through induction of EMT [21, 34, 35]. Here, we present convincing evidence that vimentin is an important mediator of OPN-induced effects on EMT. Effects on some EMT marker expression by OPN were abolished by vimentin inhibition, strongly supporting that OPN stimulated effects can be mediated by vimentin. Previous *in vitro* experiments have hinted at an active role for Axl–a novel downstream effecter of vimentin in regulating EMT of tumor cells, in tumor biology [21]. We also revealed alteration of OPN expression in HCC cells was accompanied by corresponding changes in Axl protein levels. Therefore, we propose that OPN induces EMT and promotes metastasis of HCC cells, at least in part, by up-regulating vimentin and then influencing Axl expression.

In summary, in this study, we reveal a novel mechanism that OPN induces EMT through binding to vimentin to promote HCC metastasis. Uncovering a novel function and molecular mechanism for OPN will shed new light on the understanding of tumor progression and open tantalizing therapeutic possibilities in HCC.

## MATERIALS AND METHODS

### Selection of patients and clinical specimens

A total of 384 patients, who underwent curative liver resection for HCC at Liver Cancer Institute, Fudan University (Shanghai, China) between 2004 and 2006, were enrolled in this study. The diagnosis of HCC was confirmed by two pathologists. The patients did not receive any neo-adjuvant or adjuvant treatment.

Frozen tissue samples were collected from 10 HCC patients who had a solitary primary tumor and intra-hepatic spreading or PVTT, and used in qRT-PCR studies. Tissue samples were collected immediately after resection, transported in liquid nitrogen, and stored at −80°C until use. Formalin-fixed and paraffin-embedded tissues from 374 consecutive HCC patients were used to construct a tissue microarray (TMA) for immunohistochemistry studies. This study was approved by the Research Ethics Committee of Zhongshan Hospital, Fudan University. Informed consent was obtained according to the committee's regulations and the Declaration of Helsinki. The data did not contain any information that could lead to patient identification.

All patients were followed up until May 2013, with a median follow-up of 62.7 months (range 2–110 months). During the follow-up, patients were monitored every 2–3 months after operation. Liver function, α-fetoprotein (AFP), and hematological parameters were examined, and liver ultrasonography was done by independent doctors who had no knowledge of this study. If recurrence was suspected, CT scan or MRI was performed immediately. A diagnosis of recurrence was based on typical imaging appearance and/or an elevated AFP level. The detailed clinicopathological characteristics are summarized in Supplementary Table 1.

### Plasmids and cell lines

Five human HCC cell lines with various metastatic potentials (HepG2, Hep3B, MHCC97-L, MHCC97-H and HCC-LM3), and embryonic kidney cell line HEK 293T were used in this study. MHCC97-L, MHCC97-H, HCC-LM3 were established from the same parent human HCC cell line, at the authors^'^ institution. They have a genetically identical background and stepwise increasing metastatic potentials. HepG2 and Hep3B cells were purchased from the Shanghai cell bank, Chinese Academy of Sciences. These cell lines were routinely maintained in Dulbecco's modified Eagle's medium (DMEM) (Gibco BRL, Grand Island, USA) supplemented with 10% (v/v) fetal bovine serum (FBS) (Gibco BRL) at 37°C in a humidified incubator containing 5% CO_2_.

Full-length OPN and vimentin cDNAs were cloned into the downstream of tag in Lentiviral vectors pWPI.1. Full-length and different Vimentin truncates were also cloned into pEGFP-C3-His vector. OPN shRNA, vimentin shRNA and non-target shRNA control (pLKO.1 TRC, Mission RNAi) constructs were from SIGMA (SIGMA, Saint-Quentin Fallavier, France). All these constructs and oligonucleotides were transfected into HCC cells using Lipofectamine 2000 according to the product manual (Invitrogen).

### TMA and immunohistochemistry (IHC)

Formalin-fixed and paraffin-embedded tissues were used to construct TMA (in collaboration with Shanghai Biochip Company Ltd, Shanghai, China). Immunostaining was performed on tissue microarray slides using a two-step protocol according to the manufacturer's instructions. Briefly, following deparaffinization, the tissue slides were rehydrated and subjected to antigen retrieval by microwaving in 0.01 mol/L sodium citrate (pH 6) for 10 minutes; and then were incubated at 4°C overnight with the monoclonal antibodies against OPN (Abcam, Cambridge, UK), Twist1 (Abcam), E-cadherin(Epitomics, CA, USA), N-cadherin(Millipore, Massachusetts, USA), Vimentin (Abcam). Immunostaining was performed using ChemMate DAKO EnVision Detection Kit, Peroxidase/DAB, Rabbit/Mouse (Dako Cytomation, Glostrup, Denmark), according the manufacturer's instructions. Subsequently, they were counterstained with hematoxylin (DAKO) and mounted in dimethyl benzene. Negative controls were included in all assays and treated identically but with the primary antibodies omitted. The staining intensity was scored manually (0, no staining; 1, weak; 2, moderate; 3, strong) by two independent experienced pathologists. Five fields of cancer cells were randomly selected for scoring based on the proportion of positively stained cells (0–100%). The final IHC scoring was performed from the multiplication between intensity and proportion scores of positive cells. Expression levels of OPN and vimentin in all 374 samples were quantified. Stained tissue sections were evaluated by an expert pathologist and a scientist without knowledge of other characteristics of the samples.

### RNA isolation, reverse-transcription, and qRT-PCR

Total RNA was extracted from cell lines and frozen tumor specimens using Trizol Reagent (Invitrogen, CA, USA). Total RNA (1 μg) was reverse transcribed using PrimeScript^®^ reverse transcriptase Master Mix (TaKaRa, Dalian, China) reverse transcriptase according to the manufacturer's instructions. For qRT-PCR analysis, aliquots of cDNA were amplified using SYBR Premix Ex Taq (TaKaRa). The following primers were: forward, 5-CA GCCACAAGCAGTCCAGATTAT-3; reverse, 5-CTTT TGGGGTCTACAACCAGCATA-3 for OPN, forward, 5-AGTCCACTGAGTACCGGAGAC-3; reverse, 5-CAT TTCACGCATCTGGCGTTC-3 for vimentin, forward, 5-CTCATGGACTAATTATGGACAGGA-3; reverse, 5-TT GACTGGTCATTACAATAGCTCTT-3 for HPRT. PCR reactions were done in triplicates with following conditions: 95°C/30s, 35 cycles of 95°C/5s, 60°C/15s and 72°C/10s using the ABI PRISM^®^ 7900HT Sequence Detection System (Applied Biosystems, CA, USA) and repeated at least three times. Relative mRNA levels were normalized to HPRT, which yielded a 2^−ΔΔCt^ value as relative expression of OPN and vimentin, respectively.

### Detection of protein by western blot

The protein expression levels of OPN, Vimentin, E-cadherin, N-cadherin, Twist1, AXL and glyceraldehyde-3-phosphate dehydrogenase (GAPDH) were evaluated by Western blot. Total protein was extracted from cells by lysing the cells in RIPA buffer [50 mM Tris-HCl (pH 7.4), 0.15 M NaCl, 1% NP-40, 0.25% Na-deoxydiolate, 1 mM EDTA] containing protease inhibitors (1 mM phenylmethylsulfonyl fluoride, 1 μg/ml aprotinin, 1 mM Na_3_VO_4_, 1 Mm NaF). Protein samples was separated by SDS-PAGE (sodium dodecyl sulfate–polyacrylamide gel electrophoresis) and transferred onto PVDF (polyvinylidene fluoride) membranes. After blocked with 5% non-fat milk/TBST, the membrane was incubated with the primary antibody. The following primary antibodies were used: anti-OPN (Abcam, Cambridge, UK), anti-Vimentin (Abcam), anti E-cadherin (Cell Signal Tech, Danvers, USA), anti-N-cadherin (Millipore, Massachusetts, USA), anti-Twist1 (Abcam), anti-Snail (Cell Signal Tech), anti-AXL (Abcam), or anti-GAPDH (Cell Signal Tech); and detected with enhanced chemiluminescence reagents (Thermo Fisher Scientific). Bands were acquired by Molecular Imager ChemiDox XRS+ Imaging System with Quantity One Image software (Bio-Rad Laboratories).

### *In vitro* migration and invasion assays

Wound healing assays were used to evaluate the migration ability of cells tested. Monolayers of transfected cells were plated in 24-well plates. Cell layer was scratched with the tip of a 200 μL pipette and rinsed several times with medium to remove dislodged cells. Cells that had migrated into the wound area were photographed at the 48th hour after scratching.

Matrigel transwell assays were used to evaluate the cell invasion abilities. 5 × 10^4^ cells were plated into the upper chamber of a polycarbonate transwell filter chamber coated with Matrigel (BD) and incubated for 48 hours. Cell counts are expressed as the mean number of cells per field of view. All experiments were performed in triplicate.

### *In vivo* assays for tumor growth and metastasis

All experimental procedures involving animals were approved by The Animal Care and Use Committee of Fudan University, China. HepG2 (5 × 10^6^) transfected with OPN expression vector or control vector were implanted subcutaneously into the flank of nude mice (BALB/c nu/nu, 4 weeks). Tumor growth was monitored with tumor volume, which was calculated as previously described [36]. The mice were sacrificed 6 weeks later, and the liver and lung metastases were assessed using the NightOWL LB981 bioluminescence imaging system (Berthold Technologies, Bad Wildbad, German). The emission spectrum is filtered using anHQ520 bandpass filter (Chroma Technology) to enhance the GFP fluorescence relative to the autofluorescence signal from endogenous tissue. The liver and lung of the mice were placed in the light-tight chamber, and imaged with an exposure of 100 milliseconds. Alterations in fluorescent signals were depicted on a graph. Images were acquired and processed using WinLight32 software.

HepG2-OPN cells (5 × 10^6^) transfected with shVimentin or scrambled shRNA were implanted subcutaneously into the flank of nude mice (BALB/c nu/nu, 4 weeks). The mice were sacrificed 6 weeks later, and the tumors and lungs were removed, fixed in formalin, and embedded in paraffin. Consecutive sections were made for every tissue block of the lung and stained with hematoxylin-eosin (H & E). Lung metastases were examined and classified.

OPN, E-cadherin, vimentin, Twist and N-cadherin *in vivo* expression in excised tumors were examined in tissue sections.

### IP assays

Cells were washed twice with ice-cold PBS and then lysed with RIPA lysis buffer supplemented with complete protease inhibitor (Roche Applied Science). The lysate was cleared by centrifugation at 12000 g before being loaded to M2 anti-Flag mAb agarose beads (Sigma, St. Louis, MO) pre-equilibrated in RIPA buffer overnight at 4°C. The beads were washed with RIPA buffer five times and bound proteins eluted using Flag peptide (Sigma). The washed protein was boiled in loading buffer, resolved on SDS-PAGE. Subsequent immunoblots were probed with the appropriate antibody and detected by ECL.

### MS

Immunoprecipitation was performed as described above. The immunoprecipitates with Flag-beads were resolved on SDS-PAGE denaturing gel, visualized by Coomassie blue staining, and the protein band of interest was removed for MS analysis. MS was performed under a nano Acquity UPLC system (Waters Corporation, Milford, USA) connected to an LTQ Orbitrap mass spectrometer (Thermo Electron, Bremen, Germany) equipped with an online nanoelectrospray ion source (Michrom Bioresources, Auburn, CA).

### Immunofluorescence assay and confocal immunofluorescence

Cells were permeabilized with 0.1% Triton X-100 for 10 min at room temperature, washed with PBS, and blocked with PBS containing 1% bovine serum albumin (BSA) for 1 h at room temperature. Cells were treated with antibody overnight at 4°C. Cells were rinsed with PBS and then incubated with secondary antibody for 1 h at room temperature. The slices were counterstained with diamidino phenylindole (DAPI) and examined using confocal fluorescence microscopy (Nikon, Tokyo, Japan).

### *In vivo* ubiquitination assay

MHCC-97L cells with stable overexpression of OPN or empty control transfected with FLAG-tagged vimentin. 24 hours after transfection, cells were incubated with 40 μM MG-132 for 36 hours, then washed twice with PBS. Cell were extracted in 2 ml RIPA lysis buffer supplemented with complete protease inhibitor (Roche Applied Science). Next, 20 μl M2 anti-Flag mAb agarose beads (Sigma, St. Louis, MO) was used to immunoprecipitate FLAG-vimentin protein in the cell extract at 4°C for 3 hours. The beads were washed with RIPA buffer five times and bound proteins eluted using Flag peptide (Sigma). The washed protein was boiled in loading buffer, resolved on SDS-PAGE for detection of vimentin and ubiquitin by Western blot.

### *In vitro* binding assay

For purifying Flag-tagged fusion OPN proteins, HEK-293T cells with stable overexpression of FLAG-OPN were harvested with 2 ml RIPA lysis buffer supplemented with complete protease inhibitor (Roche Applied Science). The lysate was cleared by centrifugation at 12000 g before being loaded to M2 anti-Flag mAb agarose beads (Sigma, St. Louis, MO) pre-equilibrated in RIPA buffer overnight at 4°C. The beads were washed with RIPA buffer five times and bound proteins eluted using Flag peptide (Sigma). The washed buffer containing Flag-OPN proteins were stored at 4°C for the next experiment. For purifying His-EGFP-tagged full-length or truncated Vimentin proteins, HEK-293T cells were transfected with His-EGFP-tagged full-length or truncated Vimentin vectors. After the transfection for 72 hours, cells were harvested with 10 ml RIPA lysis buffer supplemented with complete protease inhibitor (Roche Applied Science). His-EGFP-tagged full-length or truncated Vimentin proteins were then purified with a Ni-NTA system (7seapharmtech, China). Then, 2 μg of FLAG-OPN protein was incubated with approximately the same amount of His-EGFP-tagged full-length or truncated Vimentin proteins in binding buffer containing 0.2% Triton X-100, 50 mM Tris•Cl (pH 7.5), 100 mM NaCl, 15 mM EGTA, 1 mM DTT supplemented with complete protease inhibitor. Protein complex was pulled down with Anti-His Affinity Resin (GenScript, China), washed four times with washing buffer [0.5% Triton X-100, 50 mM Tris•Cl (pH 7.5), 100 mM NaCl, 15 mM EGTA, 1 mM DTT] supplemented with complete protease inhibitor, and then subjected to Western blot analysis.

### Cytotoxic effects of cycloheximide

The MHCC-97L with stable overexpression of OPN or empty control were seeded (40,00 cells) in 96-well dishes. After 24 hr, cells were cultured and exposed to 20 μg/mL cycloheximide (CHX) solutions. DMSO was used as control. After exposing the cultured cells to the DMSO and CHX solutions for 0 h, 2 h, 4 h, 6 h or 8 h, cell metabolic activity was measured by MTT assay at different time points.

### Statistical analyses

Statistical analyses were performed with SPSS 15.0 (SPSS Inc., Chicago, IL). Values were expressed as the mean ± standard deviation. Fisher's exact tests and χ2 tests were applied to compare qualitative variables; and quantitative variables were analyzed by the *t* test, paired Wilcoxon signed-rank test or Spearman rank correlation test. All statistical tests were two-sided, and *P* < 0.05 was considered statistically significant.

The Kaplan-Meier method was used to calculate the survival and recurrence curves and the significance was determined by the log-rank test. For OPN or vimentin density, the cutoff for the definition of subgroups was the median value. Samples were then separated into two groups for each analysis. The first group was comprised of HCCs with OPN and/or vimentin level over the median value, and the second group comprised the rest ones; each data set was analyzed separately.

## SUPPLEMENTARY MATERIALS FIGURES AND TABLE


