# Supplementary material for: Osteopontin promotes epithelial-mesenchymal transition of hepatocellular carcinoma through regulating vimentin
Source: Oncotarget. 2016 Jan 25;7(11):12997–3012. doi: 10.18632/oncotarget.7016 (PMC4914337; doi:10.18632/oncotarget.7016)
Supplement: Supplementary file 1 [file oncotarget-07-12997-s001.pdf]

Osteopontin promotes epithelial-mesenchymal transition of hepatocellular carcinoma through regulating vimentin

Supplementary Materials

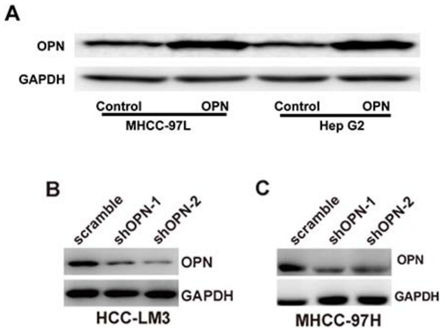

Supplementary Figure S1: OPN protein levels in HCC cells detected by western blot. (**A**) Overexpression of OPN protein levels in MHCC-97L and HepG2 cells detected by western blot. B,C. The knockdown levels of OPN protein in HCC-LM3 (**B**) and MHCC-97H (**C**) cells detected by western blot.

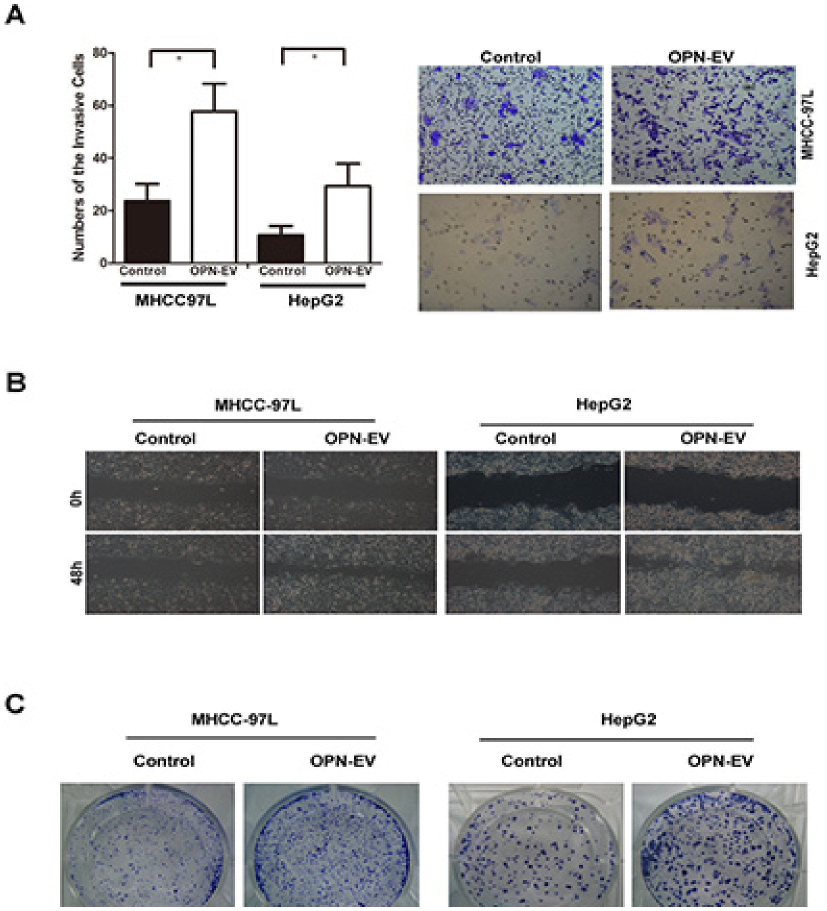

Supplementary Figure S2: The effects of OPN up-regulation on the *in vitro* invasion and migration of HCC cells. The *in vitro* (**A**) invaded cell numbers, (**B**) wound healing rates (**C**) and colony formation activity of the OPN- MHCC-97L and OPN-HepG2 cells were significantly increased compared with the controls. *$P < 0.05$.

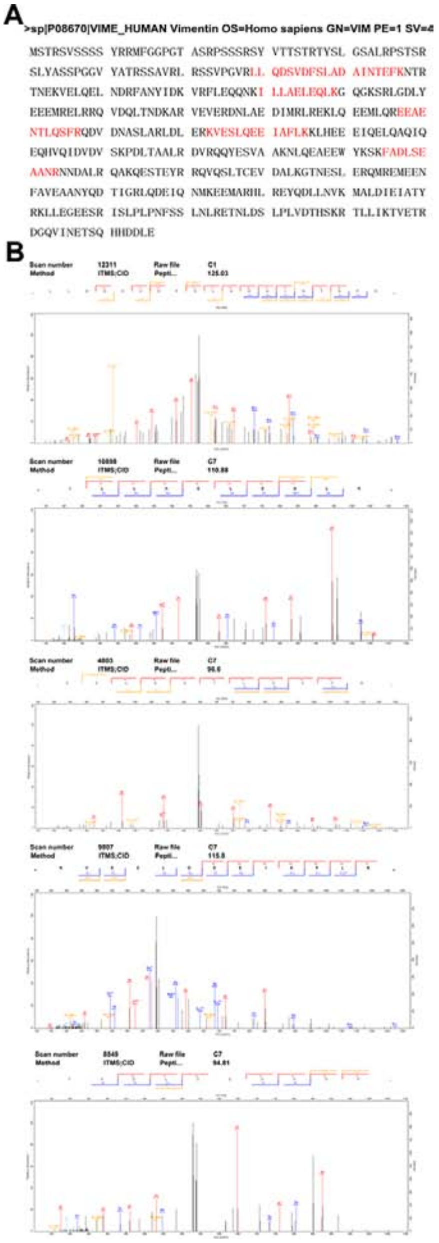

Supplementary Figure S3: MS results of vimentin protein identification. (**A**) Identified sequences (Red) covered 13.5% of the full-length vimentin protein sequence. (**B**) Mass spectra of selected sequences.

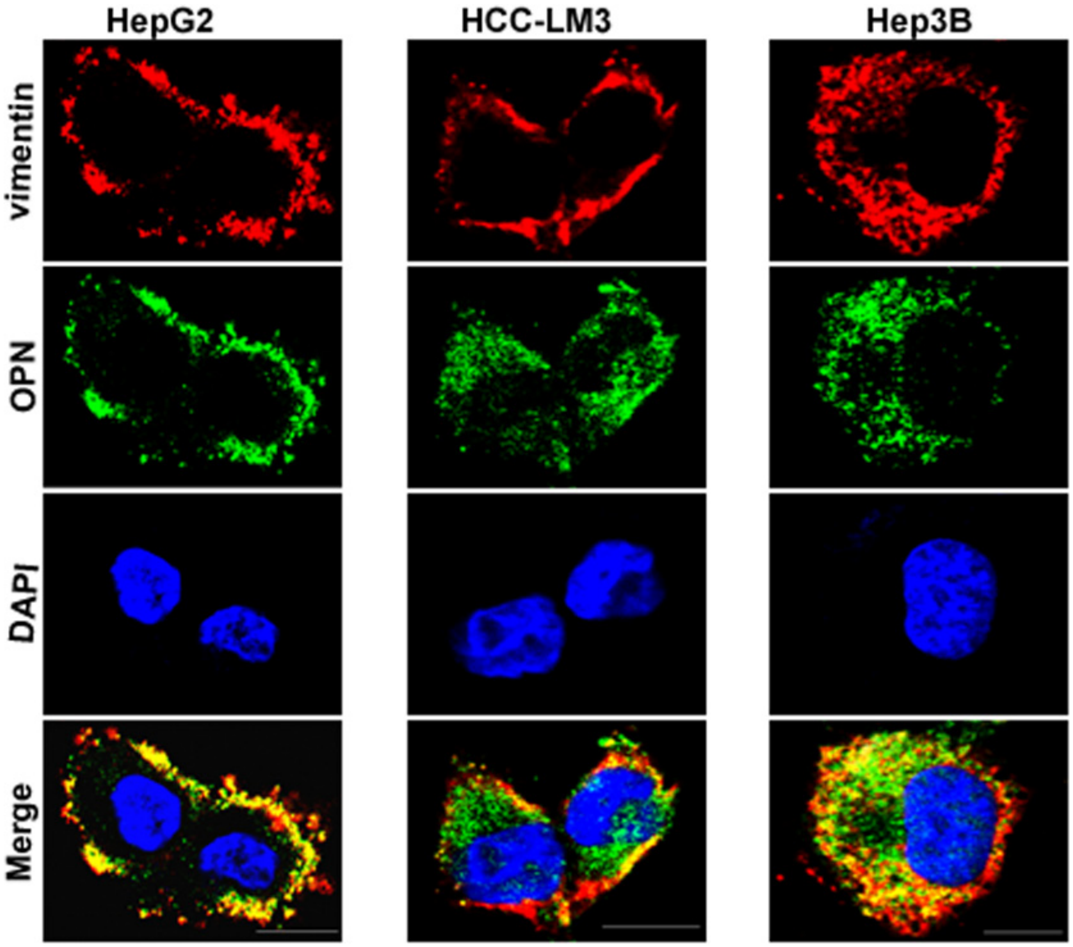

Supplementary Figure S4: Colocalization of OPN (green) and vimentin (red) in HCC cells by immunofluorescence. Nuclei were stained with DAPI. (Bar = 10 μm).

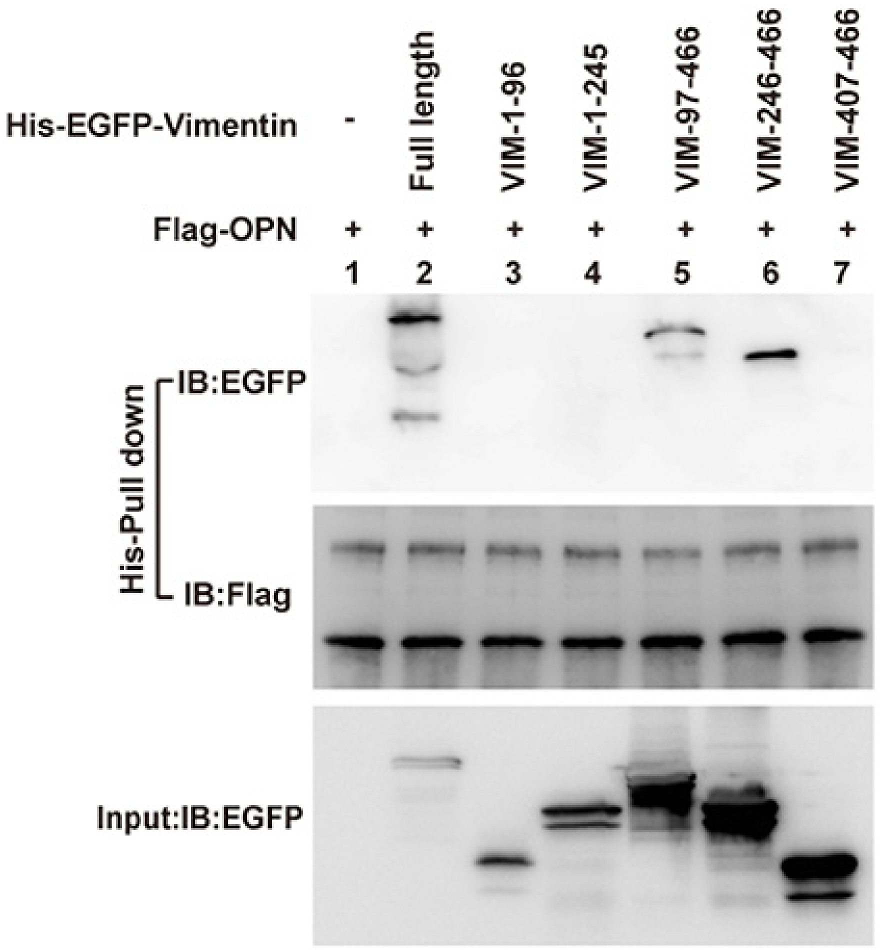

Supplementary Figure S5: Vimentin binds to OPN by *in vitro* binding assay. *In vitro* binding assay show that full-length and 246–466 fragment of Vimentin binds to OPN. The inputs are 2% of total purified proteins.

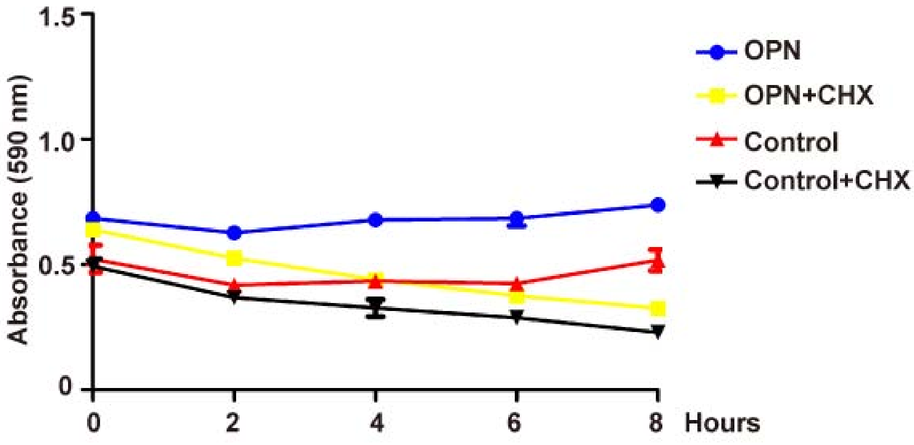

Supplementary Figure S6: Cytotoxic effects of cycloheximide at different time points on MHCC-97L cell lines stably transfected with OPN or empty vector.

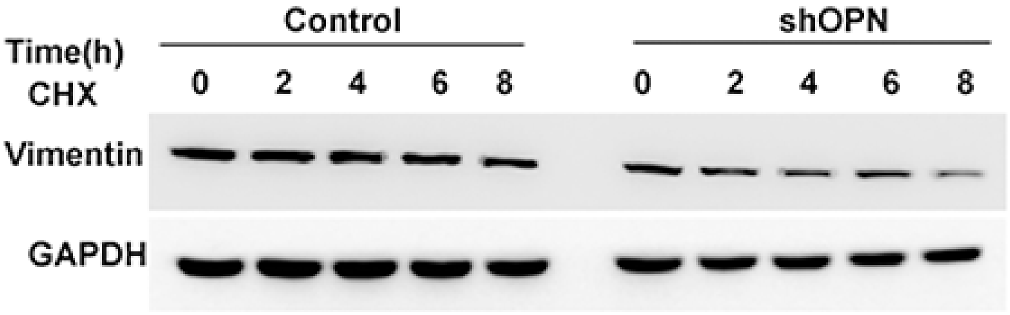

Supplementary Figure S7: MHCC-97H cells with stable knockdown of OPN were treated CHX (20 µg/mL) for indicated times. Endogenous vimentin protein levels were determined. MHCC-97H cells were stably transfected with empty vector used as control.

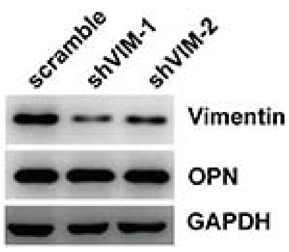

Supplementary Figure S8: The knockdown levels of vimentin protein in HepG2-OPN cells detected by western blot.

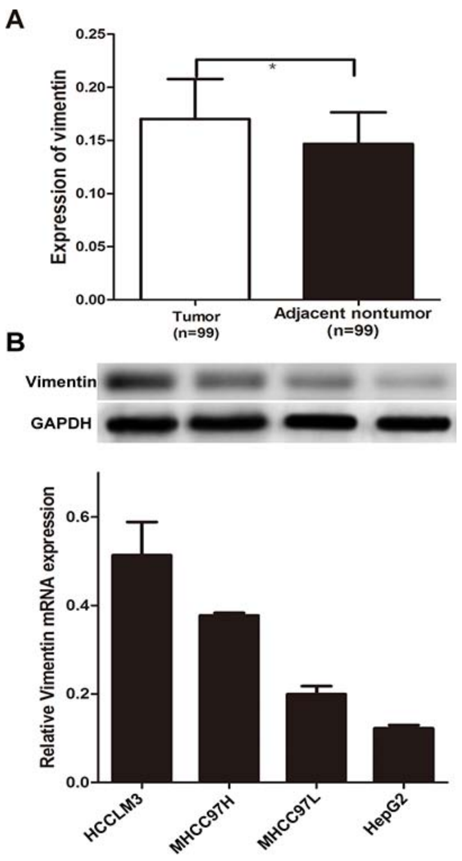

Supplementary Figure S9: The expression levels of vimentin in tumor tissues from patients with HCC and HCC cell lines. (**A**) Expression of vimentin levels in primary tumors and their corresponding adjacent nontumorous liver tissues of patients with HCC. (**B**) The mRNA (upper) and protein (lower) levels of vimentin in HCC cell lines with different metastatic potentials examined by qRT-PCR and Western blot. **P* < 0.05. Bars = mean ± S.D., *n* =3.

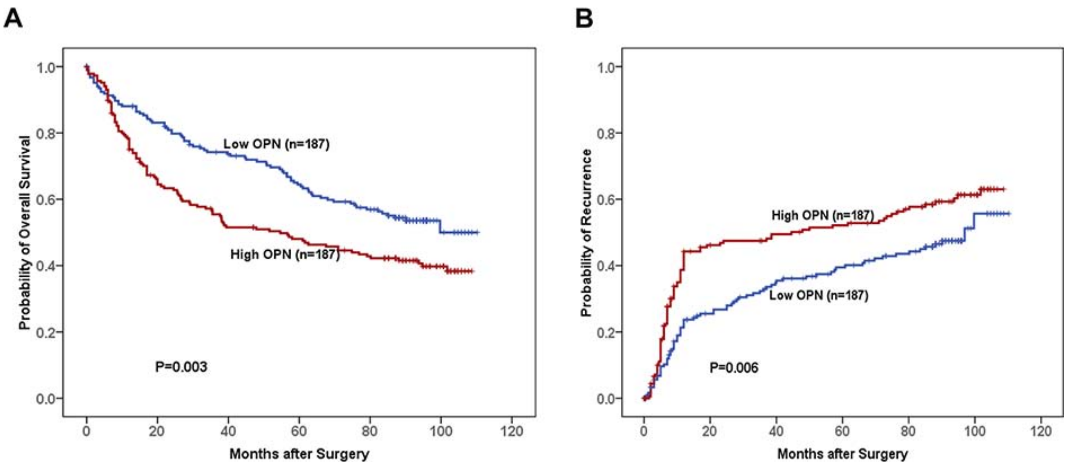

Supplementary Figure S10: The expression levels of OPN in HCC tissues. A,B. The differences in (**A**) overall survival rates and (**B**) possibilities of tumor recurrence between two groups of patients with different OPN expression levels after HCC resection. A “low” versus “high” OPN expression was defined according to the cut-off values of OPN level which were defined as the median of the cohort.

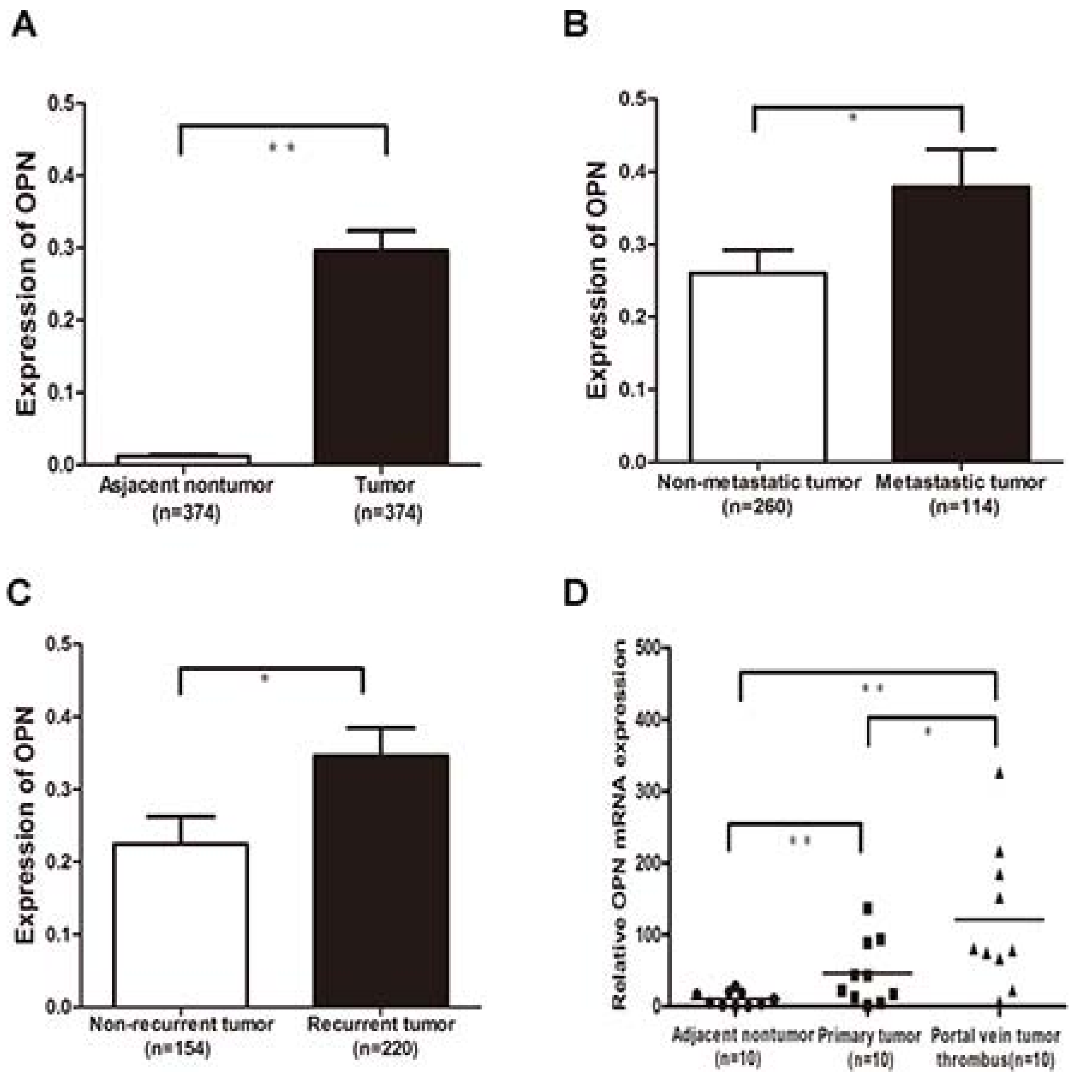

Supplementary Figure S11: The expression levels of OPN in HCC tissues. A,B,C. Comparing differences in the protein levels of OPN between (**A**) tumor and corresponding non-tumor tissues, (**B**) metastatic and non-metastatic HCC tissues, (**C**) tumor tissues from recurrent and non-recurrent groups. (**D**) Comparison of OPN mRNA levels in 10 pairs of HCC tissue samples between adjacent nontumorous liver tissues, primary HCC and their portal vein tumor thrombus. *$P < 0.05$, **$P < 0.01$.

Supplementary Table S1: The Clinicopathological characteristics of 374 patients with HCC

Characteristics	No. of Patients (%) (*n* = 374)
Age—yr	
Median	51.87
Interquartile range	22~76
Male sex — no. (%)	306 (81.8)
HBV infection — no. (%)	348 (93.0)
Tumor number > 1 — no. (%)	18 (4.8)
Tumor diameter — cm	
Median	4.22
Interquartile range	1.0~29
Vascular invasion — no. (%)	114 (30.5)
Alpha-fetoprotein > 20 ng/ml — no. (%)	236 (63.1)
Liver cirrhosis — no. (%)	332 (88.8)
Tumor encapsulation — no. (%)	173 (46.3)
Tumor differentiation — no. (%)	
I~II	280 (74.9)
III~IV	94 (25.1)
BCLC stage — no. (%)	
0 and A	97 (25.9)
B and C	277 (74.1)

Note : BCLC stage, Barcelona Clinic Liver Cancer stage.
